# Pathophysiological Role of Extracellular Purinergic Mediators in the Control of Intestinal Inflammation

**DOI:** 10.1155/2015/427125

**Published:** 2015-04-07

**Authors:** Yosuke Kurashima, Hiroshi Kiyono, Jun Kunisawa

**Affiliations:** ^1^Laboratory of Vaccine Materials, National Institute of Biomedical Innovation, Osaka 567-0085, Japan; ^2^Division of Mucosal Immunology, Department of Microbiology and Immunology, The Institute of Medical Science, The University of Tokyo, Tokyo 108-8639, Japan; ^3^Core Research for Evolutional Science and Technology, Japan Science and Technology Agency, Tokyo 102-0075, Japan; ^4^International Research and Development Center for Mucosal Vaccines, The Institute of Medical Science, The University of Tokyo, Tokyo 108-8639, Japan; ^5^Department of Microbiology and Immunology, Kobe University School of Medicine, Kobe 650-0017, Japan; ^6^Graduate School of Pharmaceutical Sciences and Graduate School of Dentistry, Osaka University, Osaka 565-0871, Japan

## Abstract

Purinergic mediators such as adenosine 5′-triphosphate (ATP) are released into the extracellular compartment from damaged tissues and activated immune cells. They are then recognized by multiple purinergic P2X and P2Y receptors. Release and recognition of extracellular ATP are associated with both the development and the resolution of inflammation and infection. Accumulating evidence has recently suggested the potential of purinergic receptors as novel targets for drugs for treating intestinal disorders, including intestinal inflammation and irritable bowel syndrome. In this review, we highlight recent findings regarding the pathophysiological role of purinergic mediators in the development of intestinal inflammation.

## 1. General Features and Metabolism of ATP in the Intestinal Compartment

Damage, trauma, and pathogenic infection cause inflammatory responses in tissues. Clinical pathologic responses involve the release of a series of inflammatory mediators, including cytokines (e.g., IL-1*β*, IL-6, and TNF*α*), lipid mediators (e.g., leukotrienes, platelet activating factor, and prostaglandins), and chemical mediators (e.g., histamine).

Accumulating evidence clearly demonstrates the importance of purinergic mediators, especially adenosine 5′-triphosphate (ATP), in the development of various inflammatory disorders [1]. In general, ATP is generated during glycolysis and the tricarboxylic acid cycle in the intracellular compartment and acts as an energy source. However, ATP is occasionally released into the extracellular compartment as so-called extracellular ATP (eATP). Biological roles of eATP were first reported in synaptic neurotransmission and neuromodulation [2]. eATP is released from nerves as a transmitter or cotransmitter and causes pain [2]. In the intestine, purinergic signaling is important for synaptic transmission in the enteric nervous system [2]. The excitatory postsynaptic potential of myenteric neurons is mediated by eATP together with nicotinic acetylcholine [3, 4]. Thus, stimulation by eATP is important for maintaining physiological intestinal motility.

In addition to nerve cells, dead, activated, or infected cells release eATP, recruiting and activating both innate and acquired immunity [5]. For instance, bacterial stimulation leads to eATP release from monocytes and enhances the production of cytokines in an autocrine manner [6]. Some gap junction hemichannels, such as pannexin and connexin hemichannels, are important for ATP release during cell activation [7]. In the steady state intestine some commensal bacteria also have the potential to release eATP [8]; thus, germ-free mice have lower luminal ATP levels than do specific pathogen-free mice. This commensal-derived eATP stimulates CD70^+^ CD11c^low^ cells in the intestinal compartment and recruits Th17 cells into the colon [9].

**Figure 1:**
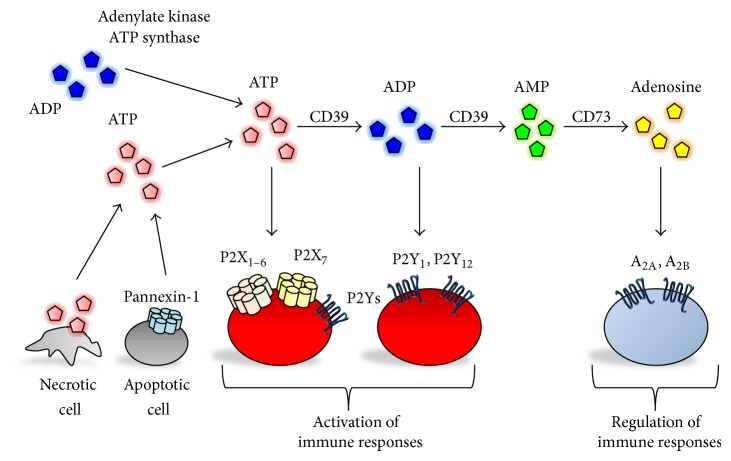
ATP is released from necrotic and apoptotic cells as extracellular ATP (eATP). Also, adenylate kinase and synthase mediate the generation of ATP in the extracellular compartment. Extracellular purines (e.g., ATP and ADP) stimulate their receptors and modulate various biological processes. Once eATP is released, the ATP is soon hydrolyzed to AMP and adenosine by the ectonucleotidases CD39 and CD73. Adenosine binds to adenosine receptors (e.g., A_1_, A_2A_, A_2B_, and A_3_).

Hydrolysis of the released eATP is catalyzed by cell surface-located enzymes, such as ectonucleoside triphosphate diphosphohydrolase family enzymes (e.g., e-NTPDase I (CD39), ectonucleotidase, and NT5E (CD73)). Consistent with the activity of eATP in the induction of intestinal Th17 cells, a deficiency of eATP-degrading enzymes elevates the concentration of luminal eATP and subsequently enhances the generation of Th17 cells in the gut [10]. By the sequential enzymatic activity of CD39 and CD73, eATP is hydrolyzed to adenosine in the extracellular compartment [11] (Figure 1). Finally, adenosine is metabolized by two pathways: one is intracellular uptake by equilibrative nucleoside transporters and the other is metabolism to AMP or inosine by adenosine kinase and adenosine deaminase, respectively [11].

Recognition of eATP is mediated by purinergic receptors, which comprise P2X (P2X_1–7_) and P2Y receptors (P2Y_1,2,4,6,11–14_). P2X_1–7_ receptors are ATP-gated ion channels and are specific for ATP, whereas P2Y receptors are G protein-coupled receptors that are specific for ADP, UTP, and ATP [5]. Each eATP-specific purinergic receptor requires a different concentration of eATP for activation. For instance, activation of P2X_7_ receptors requires a high concentration (mM level) of eATP, whereas other P2X receptors require lower concentrations (nM to *μ*M) [5]. In addition, heterooligomeric assembly occurs within P2X receptor subunits (e.g., P2X_1–3_, P2X_1–4_, and P2X_2–4-5_) and alters their functional properties, providing versatile signaling pathways mediated by eATP [12, 13].

Among several P2X and P2Y receptors, P2X_7_ is involved mainly in the induction of inflammatory responses. P2X_7_ uniquely has 200 amino acid residues in its C-terminus, which is longer than that of other P2X receptors [14]. C-terminal residues are important for receptor localization at the cell surface [14]. Stimulation of P2X_7_ by prolonged high concentrations of eATP induces pore formation in the cell membrane and increases membrane permeability [14, 15]. These pores allow influx and efflux of particles with molecular masses of up to 800 Da [11]. These changes also mediate the production of reactive oxygen species and activate inflammasome, a key molecule in the production of inflammatory cytokines such as IL-1*β* and IL-18 [5] that is responsible for inducing inflammatory responses. In addition, eATP-P2X_7_ pathways are involved in molecular shedding. Molecules responsible for adhesion (e.g., CD44 and CD62L) are shed from the cell surface by P2X_7_ activation; stimulation by eATP is thus involved in cell migration [16, 17].

## 2. Role of eATP in Prevention and Development of Infectious Diseases

Some kinds of pathogens use intestinal tissues as invasion sites. Upon infection, pathogenic components from the microorganisms stimulate innate immune cells such as macrophages and neutrophils via innate receptors such as toll-like receptors (TLRs). This stimulation induces the release of eATP through pannexin-1 hemichannels and subsequently activates P2Y_2_ and P2X_7_ receptors in an autocrine or paracrine manner and enhances cytokine production [6, 18]. In microglial cells and macrophages, initial stimulation of lipopolysaccharide- (LPS-) TLR4 pathways with subsequent signaling by the P2X_7_ pathway induces Ca^2+^ influx and IL-1*β* secretion [19]. In fact, eATP-P2X_7_ pathways play important roles in eliminating intracellular pathogens. Activation of P2X_7_ by selective agonists induces effective clearance of* Toxoplasma gondii* from infected macrophages and of chlamydia from epithelial cells [20, 21]. These signals are required for protective immunity against pathogens. In addition, a recent study found that eATP production was induced by administration of vaccine adjuvant, which is required for an effective response in vaccination against infectious agents and cancer [22].

Reciprocally, the pathogenicity of some pathogens is determined by their ability to induce eATP release. For instance, enteropathogenic* Escherichia coli *induces eATP release from host cells by killing them via type III secretion systems as well as cell-permeable cystic fibrosis transmembrane conductance regulator-mediated pathways [23]. Similarly, cholera toxin from* Vibrio cholerae* is capable of inducing eATP production [24]. Another study in colon epithelial cell lines found that adenosine, a metabolite of eATP, bound to A_2B_ receptors, resulting in short-circuit current responses causing diarrhea [23, 24].

Some kinds of pathogens have unique systems that inhibit eATP release from host cells and thus prevent the spread of infection to the host's immune system. For instance, infection of epithelial cells with* Shigella flexneri* induces eATP release via connexin hemichannels in the early phase of infection, and this release alerts the host to the pathogenic infection. However, prolonged infection with* Shigella* is accompanied by the production of Ptdlns5P, a lipid mediator, to close the connexin hemichannels [25]. Another example is that of* Streptococcus agalactiae*, a commensal bacterium that resides in the intestine or vaginal mucosa but occasionally shows pathogenicity, causing neonatal pneumonia.* Streptococcus agalactiae *releases ecto-5′-nucleoside diphosphate phosphohydrolase and degrades extracellular nucleotides, including eATP; it thus turns off the eATP-mediated alerting of the host defenses to danger [26, 27].

## 3. Pathological Aspects of eATP in the Mucosal Compartment

eATP-purinergic receptor-mediated pathways are now considered to be targets for the treatment of inflammatory disorders in the systemic compartment, including inflammatory pain and rheumatoid arthritis [28]. Accumulating evidence suggests that eATP-purinergic receptor-mediated pathways are also potential targets for the treatment of inflammatory diseases of mucosal tissues in, for example, the respiratory and gastrointestinal tracts [4, 5, 29]. In the asthma model, migration of eosinophils, dendritic cells, and Th2 cells into the inflamed lung is mediated by the P2Y_2_ receptor; therefore, P2Y_2_-deficient mice show reduced inflammatory responses [30]. Th2-type immune responses are also induced by dendritic cells expressing P2X_7_. Indeed, depletion of eATP by apyrase treatment or P2X_7_ deficiency reduces signs of inflammation in the upper respiratory tract [31]. It was recently found that the functional capacity of P2X_7_ (i.e., its ability to promote pore formation) is associated with asthma risk or disease severity in humans [32]. Moreover,* in vivo* imaging analysis has revealed eATP release in the intestinal compartment and peritoneal cavity of mice with acute graft-versus-host disease (GVHD) [33]. Treatment with apyrase or with inhibitors of various purinergic receptors inhibits GVHD-associated intestinal inflammation. In this case, the eATP-P2X7 pathway activates dendritic cells and consequently induces Th1 immune responses (e.g., IFN*γ* production) and expansion of donor T cells, thus contributing to the onset of inflammation.

Several studies have revealed the pathologic roles of eATP and purinergic receptors (especially P2X_7_) in the development of intestinal disorders, including irritable bowel syndrome (IBS) and inflammatory bowel disease (IBD) [1, 34] (Table 1). IBS is a common gastrointestinal disorder characterized by discomfort, chronic abdominal pain, and altered bowel habit. Sometimes it occurs after intestinal infection. One meta-analysis has demonstrated that the risk of IBS increases 600% after gastrointestinal infection [35]. Consistently, it has been reported that transient intestinal infection with* Trichinella spiralis* in mice causes postinflammatory visceral hypersensitivity, which is associated with IL-1*β* production mediated by eATP-P2X_7_ pathways [34] (Table 1). Because mast cells are considered to play a critical role in the development of IBS and express high levels of P2X_7_, it is possible that the eATP-P2X_7_ pathway in mast cells is involved in the development of IBS [36].

The eATP-purinergic receptor pathway, especially the eATP-P2X_7_ pathway, is also involved in the development of IBD. Overexpression of P2X_7_ receptors has been observed in the intestinal mucosa of patients with IBD—especially Crohn's disease [44]. Experimentally, P2X_7_-deficient mice do not develop experimental colitis, and inhibition of P2X_7_ by A-740003, Brilliant Blue G, or KN-62 ameliorates experimental colitis by reducing the recruitment of neutrophils, T cells, and macrophages, as well as collagen deposition [44] (Table 1). The eATP-P2X7 pathway is therefore now considered to be a novel therapeutic target in the treatment of IBD [43, 44] (Table 1).

**Figure 2:**
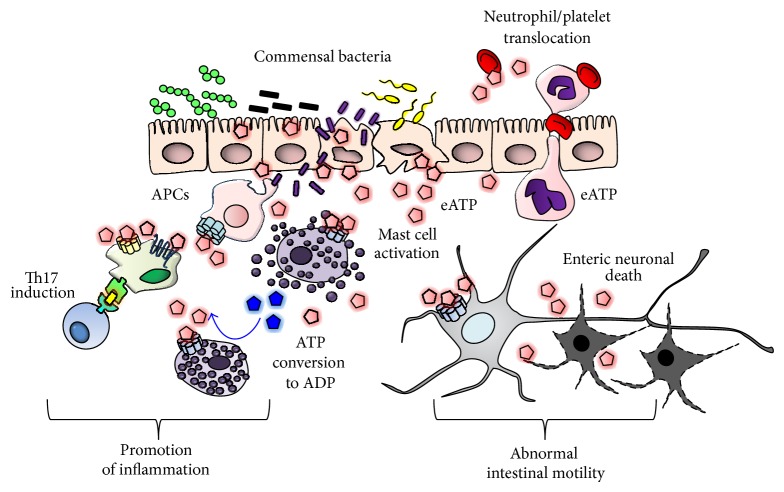
In the intestinal compartment, extracellular ATP (eATP) is released from damaged epithelial cells and commensal bacteria. Macrophages, platelets, mast cells, and neutrophils are potential source of eATP upon their activation. Neutrophils facilitate translocation of platelets across intestinal epithelium. eATP also induces Th17 cell generation, activation of mast cells, and neuronal cell death, promoting intestinal inflammation. APCs: antigen-presenting cells. eATP stimulates mast cells to induce the production of inflammatory chemokines (e.g., CCL2, CCL4, CCL7, CXCL1, and CXCL2), cytokines (IL-1*β*, IL-6, and TNF*α*), and mediators (histamines and leukotrienes).

Several mechanisms of eATP-mediated inflammation in the development of IBD have been proposed. First, eATP from damaged intestinal epithelial cells, which are frequently observed in IBD patients, and inflammatory cells (e.g., neutrophils and macrophages) stimulates dendritic cells to produce IL-6, IL-12, and IL-23 and TGF*β*, thus inducing the production of inflammatory Th1 and Th17 cells [42, 43] (Figure 2) (Table 1). Enteric neuronal cell death is frequently observed in intestinal inflammation and causes colonic motor dysfunction. The eATP–P2X7 pathway is involved in enteric neuronal cell death through the pannexin-inflammasome cascade, and thus colonic motor dysfunction during colitis is prevented by targeting these pathways [41] (Table 1). We previously established mast cell-specific antibody libraries and showed that P2X_7_ is expressed at high levels in mast cells in the colonic tissues [40]. eATP stimulates mast cells to induce the production of inflammatory chemokines (e.g., CCL2, CCL4, CCL7, CXCL1, and CXCL2), cytokines (IL-1*β*, IL-6, and TNF*α*), and mediators (histamines and leukotriene). Thus, blockade of P2X_7_ by a specific antibody (1F11 monoclonal antibody) inhibits mast cell activation in the colonic tissues and consequently prevents the development of intestinal inflammation [40] (Table 1). In this pathway, P2X_7_ expression on mast cells is important for the development of colitis, because mast cell-deficient mice reconstituted with P2X_7_-deficient mast cells show amelioration of inflammatory signs. Of clinical relevance, we have found that the number of P2X_7_^+^ mast cells is increased at sites of inflammation in Crohn's disease patients [40]. eATP is produced by injured epithelial cells and inflammatory cells, including neutrophils, via gap junction molecules such as connexin 43 [45]. It was reported that P2Y2 and P2X7 receptors are important for the migration of neutrophils and macrophages. In the inflammatory condition, neutrophils transmigrated between epithelial cells to the luminal part of the intestine. In this condition, platelets translocate along with neutrophils and released eATP at the mucosal surface (Figure 2) (Table 1). Additionally, mast cells express ectoadenylate kinase and ATP synthase to mediate the extracellular conversion of ADP to ATP, which in turn promotes mast cell activation in an autocrine and paracrine manner (Figure 2). We have recently found that, in contrast to the abundance of P2X_7_ expression on mast cells in the colon, there are limited levels of P2X_7_ expression on skin mast cells, which is regulated by skin fibroblasts [46]. Skin fibroblasts uniquely express Cyp26b1 to degrade retinoic acid within tissues or microenvironments; Cyp26b1 is responsible for inhibiting P2X_7_ expression [46]. Thus, unique tissue environments determine P2X_7_ expression on mast cells, which is a critical factor in the development of local inflammation.

## 4. Resolution of eATP-Mediated Inflammation for Maintenance of Mucosal Homeostasis

Once eATP is released, it is soon hydrolyzed to ADP, AMP, and adenosine by the ectonucleotidases CD39 and CD73; this is essential for resolving inflammatory responses (Figure 1). Indeed, CD39-deficient mice, as well as humans who have CD39 polymorphism and thus low levels of CD39 expression, have increased susceptibility to IBD [47]. Similarly, CD73 deficiency or administration of CD73 inhibitor (e.g., *α*,*β*-methylene ADP) enhances susceptibility to intestinal inflammation in mice [48–50].

Adenosine, which is derived from the dephosphorylation of eATP via CD39 and CD73 or diffuses directly from the intracellular compartment via equilibrative nucleoside transporters, binds to adenosine receptors such as A_2A_ and A_3_ receptors, which are involved in both the promotion and the resolution of inflammatory responses [51–53]. A_2A_ and A_3_ receptor expression on T cells and myeloid cells is a prerequisite for the inhibition of intestinal inflammation [54]. In fact, A_2A_ and A_3_ adenosine receptor-selective agonists (e.g., ATP-146e and IB-MECA, resp.) ameliorate intestinal inflammation by impairing the recruitment of inflammatory cells and the production of inflammatory cytokines [55, 56]. In addition, cyclosporine, salicylates, methotrexate, and sulfasalazine, which are used to treat IBD in humans, all decrease eATP levels and increase adenosine production, partly via the stimulation of CD73-dependent adenosine production [57]. Similarly, upregulation of CD39 expression induced on dendritic cells by IL-27 hampers Th1 and Th17 cell production and consequently prevents eATP-mediated inflammation [58]. All of this evidence indicates that inhibition of eATP signaling, together with the promotion of adenosine-mediated regulatory pathways by targeting receptors or ectoenzymes, would be a beneficial strategy for the treatment of intestinal inflammation.

## 5. Closing Remarks

The importance of purinergic signaling was recognized almost 70 years ago. Accumulating evidence has since revealed the underlying molecular and cellular mechanisms of purinergic signal-mediated maintenance and disruption of mucosal homeostasis. Currently, the clinical relevance of some of the drugs used to treat intestinal inflammation is explained by their regulation of eATP-adenosine balance. Additionally, drugs that target purinergic receptors have now undergone clinical trials [11]. Notably, ATP-adenosine balance, as well as receptor expression levels and the cells expressing these receptors, differs among tissues and environmental conditions. Further investigations using new technologies such as* in vivo* monitoring of eATP release [59, 60] will clarify the complex mechanisms of purinergic signal-mediated immune regulation. This in turn will provide further advances in the design of drugs for preventing and treating inflammatory diseases and maintaining immunologic health.

## Figures and Tables

**Table 1:** Recent reports indicating the critical roles of eATP in the adverse conditions of intestines (inflammatory bowel diseases and irritable bowel syndrome).

Enteric diseases	Receptors	Functions	Reference
Inflammatory bowel disease	P2R/A2BR	Enhance co-transmigration of neutrophils and platelets across intestinal epithelial cells in IBD patients. Platelets release large amount of ATP in the lumen metabolite to adenosine via CD73 and ecto-NTPDases expressed in epithelial cells. Adenosine-A2BR pathway induces electrogenic Cl-secretion with water movement to lumen.	[37]
	P2XR	T cell receptor stimulation induces ATP synthesis and release from activated T cells through pannexin-1 hemichannels. Released ATP activates T cells and produce IL-2 and proliferation in autocrine manner. Blockage of P2X receptors (oxidative ATP) impairs the development of colitis in mice.	[38]
	P2R	ATP released from commensal bacteria acts on CD70+ CD11c+ cells reside in the intestinal lamuna propria and induces Th17 cells in mice; degradation of ATP (by apyrase treatments) ameliorates colitis in mice.	[9]
	P2Y2	Increase of P2Y2 expression in epithelial cells is observed during colitis. P2Y2 stimulation induces release of prostaglandin E2 release from the cells and promotion of intestinal microtubule stabilization and mucosal reepithelization. Those pathways take part in the wound healing during colonic inflammation. Treatment with P2Y2 agonist improves recovery from colitis in mice.	[39]
	P2X7	ATP induces activation of mast cells and enhances inflammatory responses, upregulation of P2X7 in mast cells of Crohn's disease patients, anti-P2X7 antibody treatment inhibits colitis in mice.	[40]
	P2X7	Induction of enteric neuronal cell death and alteration of intestinal motility.	[41]
	P2R	ATP induces IL-6 and CXCL1 productions from epithelial cells; ATP influences the response of epithelial cells to various TLR ligands and induces inflammatory T cells by affecting DC maturations.	[42]
	P2X7	Prophylactic systemic P2X7 blockade (A740003 and brilliant blue G) reduces inflammatory cytokines in rats.	[43]
	P2X7	Upregulation of P2X7 in epithelium, macrophage, and dendritic cells of Crohn's disease patients, P2X7-deficient mice did not develop colitis.	[44]

Irritable bowel syndrome	P2X7	Induction of IL-1*β* and the development of postinflammatory visceral hypersensitivity in the *Trichinella spiralis-*infected mouse	[34]
